# Automatic cell identification and counting of leaf epidermis for plant phenotyping

**DOI:** 10.1016/j.mex.2020.100860

**Published:** 2020-03-14

**Authors:** Giacomo Mele, Laura Gargiulo

**Affiliations:** Institute for Agricultural and Forest Systems in the Mediterranean - National Research Council, ISAFOM-CNR, Via Patacca 85, 80056 Ercolano, Italy

**Keywords:** Leaf image analysis, Leaf optical microscopy, ImageJ

## Abstract

The counting of leaf epidermal cells is useful to study the plant developmental changes produced by environmental or genetic factors.

The scanning electron microscopy can be used, but it is expensive and time-consuming. Methods using optical microscopy are also available, but they still require leaves pre-treatment and manual cell identification.

We propose a quick and simple method for counting leaf epidermal cells without leaf treatments and based on automated cell identification and marking. It allows to highly improve the representativeness of leaf epidermis screening, aiming at a high-throughput plant phenotyping approach.

• The leaves are pressed between two glass slides without any pre-treatment and digital micrographs are acquired under incident light.
• Epidermal cells are automatically identified and counted by means of a “macro" of ImageJ
• The cell count obtained applying the procedure of image processing is very close to that obtainable by manual cell identification.

## Graphical abstract

**Figure:**
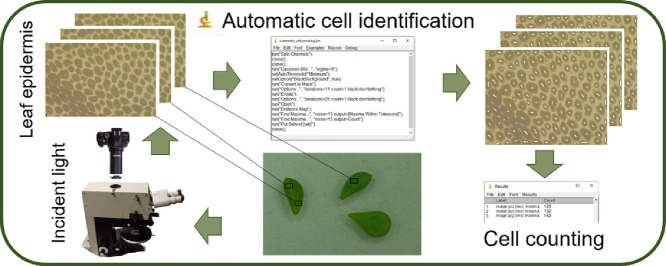

**Table:** Specifications Table

*Subject Area*	Agricultural and Biological Sciences
*More specific subject area*	*Plant Phenotyping*
*Method name*	*Leaf epidermal cell counting*
*Name and reference of original method*	J. J. Tao, S.Y. Chen, J. S. Zhang, Simple methods for screening and statistical analysis of leaf epidermal cells in dicotyledonous plants. Bio-protocol 6 (2016) 17.
*Resource availability*	*Example image files provided as supplementary material*

## Method details

### Leaf epidermal cell identification and counting procedure

#### Leaf preparation

**Fig. 1:**
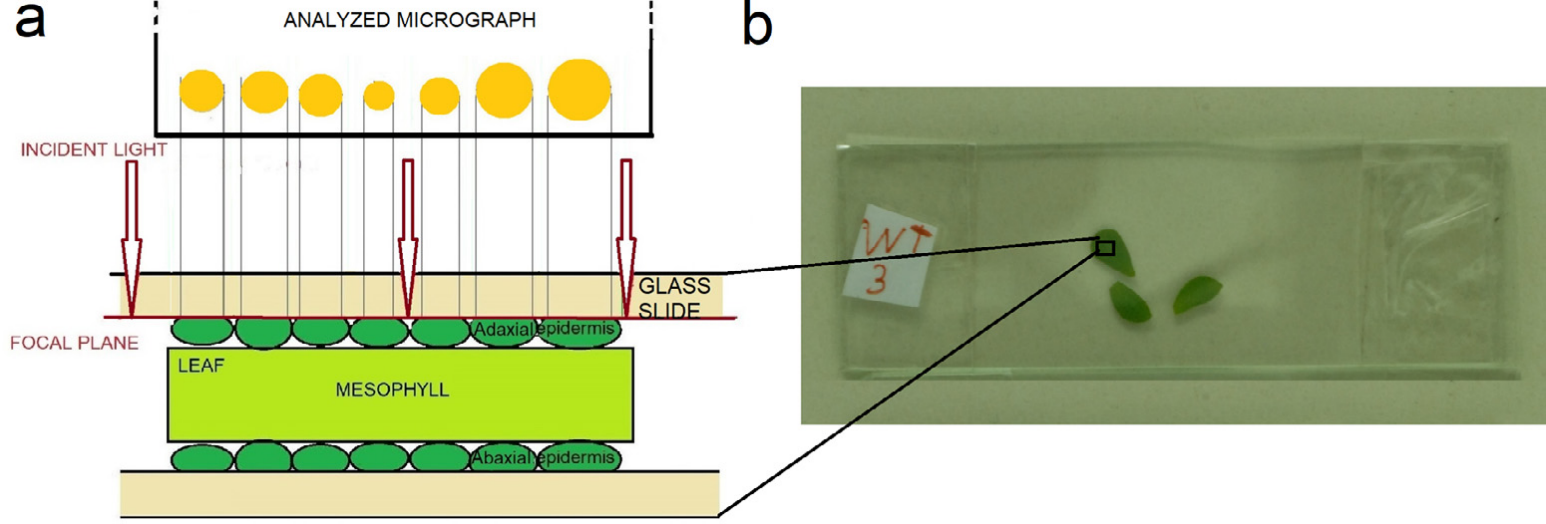
(a) Schematic representation of a part of the analyzed leaf between two glass slides, showing the contact between the cell surface and the glass. In red it is shown the focal plane position used for the image acquisition. Above there is a scheme of the analyzed image with the acquired cells (in yellow). (b) An example of three leaves of *Lotus japonicus* positioned between the pair of glass slides. (For interpretation of the references to color in this figure legend, the reader is referred to the web version of this article.)

Leaves are detached and used still fresh for the analysis. They are spread over the surface of a glass slide without any further preparation. Then they are covered with another glass slide applying enough pressure to favor the contact between the slide and the surface of all epidermal cells without crushing the cells. The most protruding part of each cell should touch the glass slides in both, adaxial and abaxial laminas (see Fig. 1(a)).The glass slides containing the leaves are placed under a visible light microscope equipped with a digital camera. The focal plane is set at the contact plane between the upper glass slide and the leaf epidermis cells (see Fig. 1(a)).

The leaves have to be sufficiently fresh for maintaining the natural turgor of epidermal cells, because the proposed procedure takes advantage of their characteristic surface convexity. Indeed, microscope focuses only on the most protruding parts of the cells that touch the glass, making they appear sufficiently separate to be correctly counted. Therefore, it is recommended to apply an adequate pressure on the glass slide with the fingers while observing the leaf under the microscope and to maintain it during micrograph acquisition. In case of large leaves with thick veins, it could be useful to cut a leaf tissue portion between the veins before to squeeze it between the glass slides.

#### Micrograph digital acquisition

A given number of microscopic fields of view (FOVs) (e.g., 0.5 mm x 0.3 mm, in Fig. 3(a)) is randomly chosen for each leaf lamina between the mid-vein and the margin of leaf. After setting a scale on the surface, the images of the FOVs are acquired under incident light by means of an SLR camera mounted on a trinocular optical microscope. As described above and shown in Fig. 1, the image acquisition is performed after focusing on the plane of contact between the cell surface and the glass slide. In this way, only the surface of each cell in contact with the glass slide appears in the acquired image.

#### Automatic cell identification and counting

Cell identification, marking and counting are automatically obtained on each micrograph applying the following operational steps of image processing: (1) extraction of the red channel, (2) filtering, (3) binarization, (4) morphological operations, (5) calculation of the distance map and (6) identification of the maxima points and counting.

**Fig. 2:**
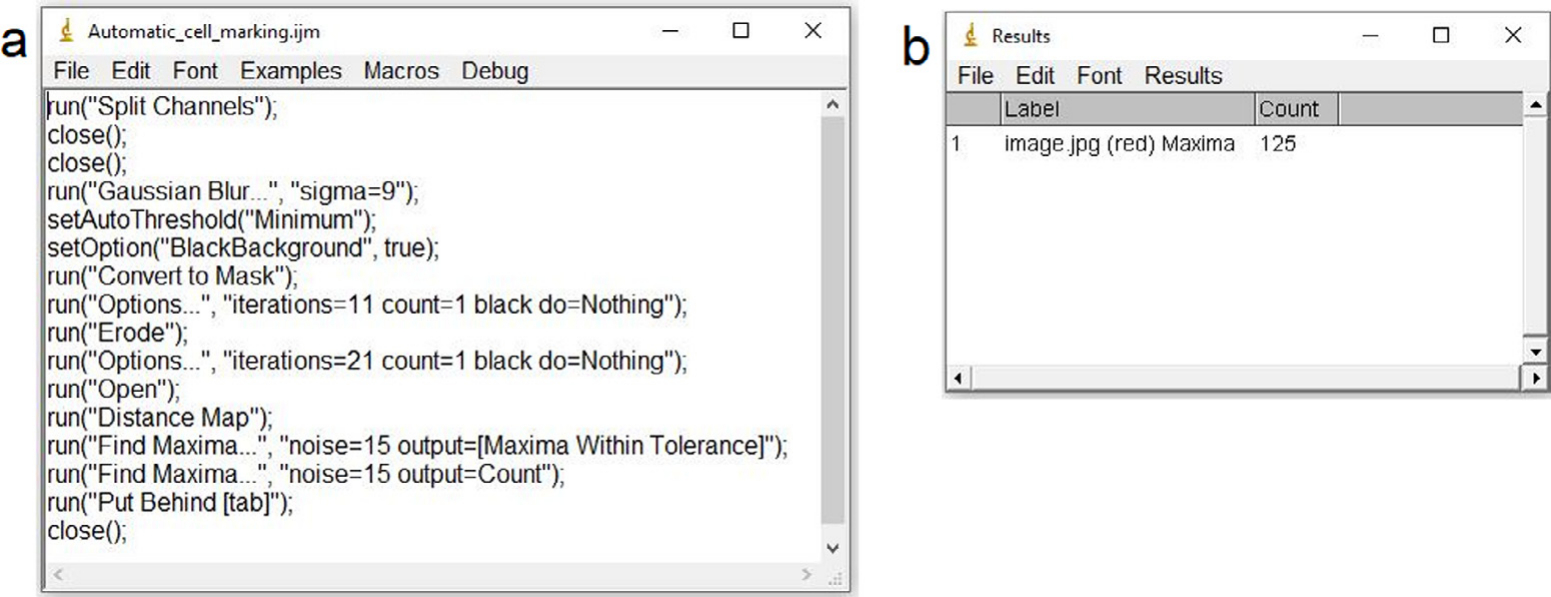
The windows of ImageJ with (a) the macro named Automatic_cell_marking and (b) the resulting cell count obtained after running the macro.

All the steps are automatically performed running the following “macro” recorded in ImageJ software [1]:

run(“Split Channels”);
close();
close();
run(“Gaussian Blur…”, “sigma=9”);
setAutoThreshold(“Minimum”);
setOption(“BlackBackground”, true);
run(“Convert to Mask”);
run(“Options…”, “iterations=11 count=1 black do=Nothing”);
run(“Erode”);
run(“Options…”, “iterations=21 count=1 black do=Nothing”);
run(“Open”);
run(“Distance Map”);
run(“Find Maxima…”, “noise=15 output=[Maxima Within Tolerance]”);
run(“Find Maxima…”, “noise=15 output=Count”);
run(“Put Behind [tab]”);
close();

Such a “macro” can be easily created in ImageJ by editing another existing macro and substituting the existing instructions with the text reported above and then saving as a new macro named e.g., “Automatic_cell_marking” (see Fig. 2).

In the following are the general rules to set the values of the parameters for the commands in the macro depending on the resolution of the analyzed micrograph.

**Fig. 3:**
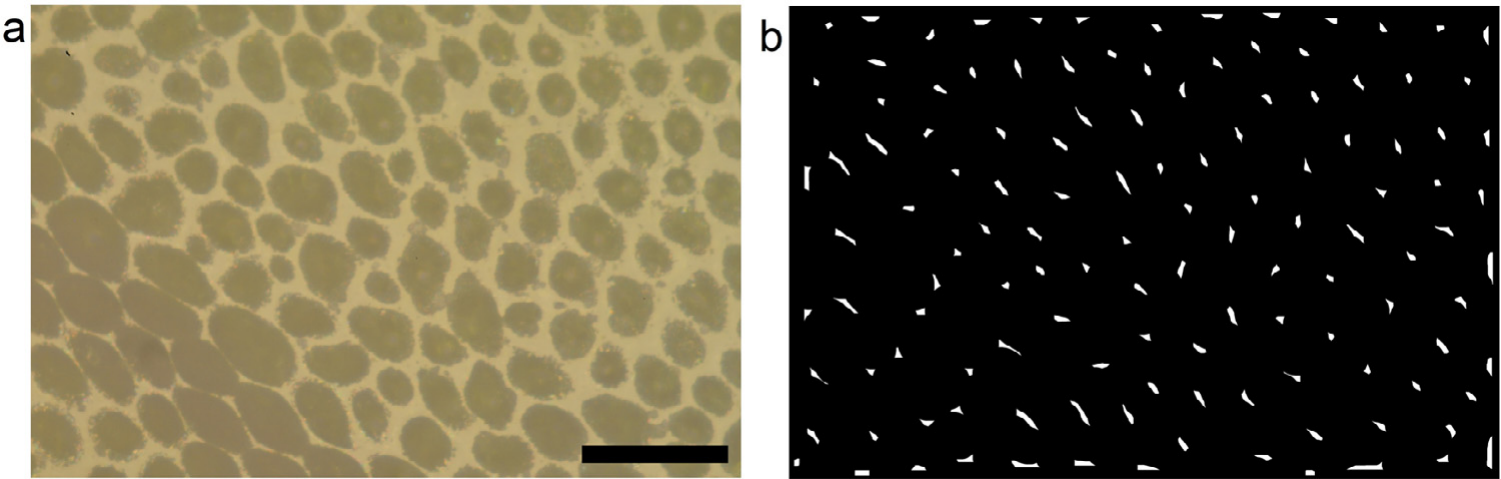
Example of (a) acquired image (micrograph of Lotus japonicus leaf) and (b) output image resulting from the “macro" of automatic marking of the cells. The black bar is 100 µm.

Given the resolution x (x=pixel size in microns), the “sigma” value of the filtering “Gaussian Blur” is 1/x; the number of iterations for the “erode” and “open” operations are equal to 1.25/x and 2.5/x, respectively. Finally, the “noise” value for the operation of “Find Maxima” is set to 1.75/x or 4.75/x, if the epidermis cells exhibit round (e.g., Fig. 3(a)) or bottleneck (e.g., Fig. 5) shaped footprint on the glass, respectively. Parameters are approximated to the nearest integer.

Values reported in the example above and in Fig. 2(a) refer to *x* = 0.117 µm and to round shaped epidermis cells (see Fig. 3(a)).

Running the above macro on the image in Fig. 3(a) chosen as an example, the output image is that in Fig. 3(b), which shows the markers automatically put on each cell. In Fig. 2(b) is shown the window of ImageJ reporting the cell counting result.

Such a macro can be run on a batch list of image files to furtherly automatize the process and to obtain highly representative results needed for phenotyping purposes, for example.

The number of cells in the FOV can be used to calculate the number of cells per leaf area unit (cell density). Furthermore, the average cell size can be determined by the ratio between the area of the FOV and the cell number.

#### Counting check (optional)

**Fig. 4:**
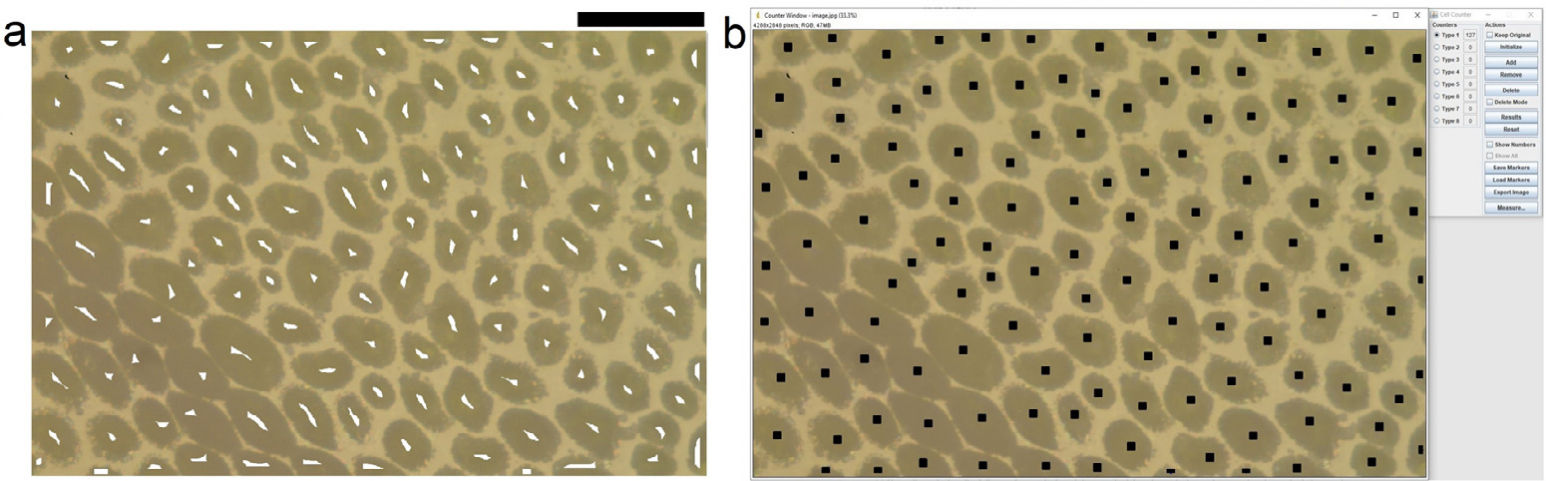
Check of the automatic counting. Comparison between (a) image resulting from the superimposition of the automatic markers (see Fig. 3b) on the example image and (b) the image obtained using the manual plugin “Cell counter” of ImageJ. The black bar is 100 µm.

At the end of the image processing, a check of the automatic cell counting result can be done on randomly chosen images: the image with the identified maxima points (automatic markers) can be added to the initial image (see resulting image in Fig. 4(a)), using the command “image calculator” of ImageJ. If more than one of the identified points correspond to a single cell, the “noise” value set for the operation of “Find Maxima” can be increased until identifying only one point for each cell.

### Comparison between automated and manual cell identification

Finally, a comparison between the automatic and manual cell identification has been performed, in order to provide a validation of the proposed method. Fig. 4(a) shows the automatically identified markers that have been superimposed to the example image of Fig. 3(a). In Fig. 4(b) markers were manually put on the cells and counted using the “Cell counter” plugin of ImageJ. It can be noted that the cells identified by the automatic procedure are the same of the manual counting, except 2 cells. Indeed the resulting cell number was 125 for the automatic procedure and 127 for the manual one. Such a slight difference is mainly due to the different way the cells cutted by the image border are considered.

**Fig. 5:**
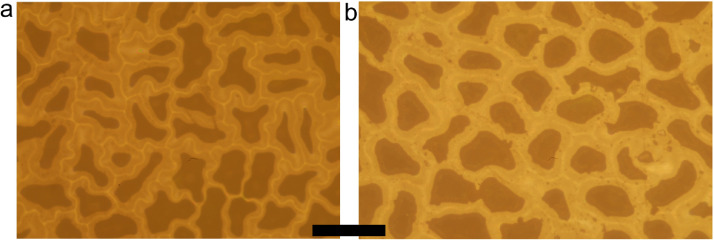
Micrographs of leaf portions of (a) *Oxalis pes-caprae* and (b) *Viola odorata*. The pixel size was 0.130 µm. The black bar is 100 µm.

The same validation procedure was repeated also on two plant species (see Supplementary Fig. 5) with larger and more irregular shaped epidermis cells, namely *Oxalis pes-caprae* (Fig. 5(a)) and *Viola odorata* (Fig. 5(b)). The cell number resulted 49 and 45 from automatic counting and 51 and 47 from manual counting for *Oxalis pes-caprae* and *Viola odorata*, respectively.

## Additional information

### Background

Morphological changes in leaf tissues and leaf expansion rates relate directly to leaf functioning in photosynthesis and transpiration. Such leaf changes are affected by both environmental and genetic factors. Leaf epidermal cell size and number are positively correlated to leaf area. Thus, leaf phenotype assessment by means of observation and statistical analysis of the leaf epidermal cells is valuable for the study of leaf development and of its response to environmental stimulus. The leaf cell counting gives also an important contribution in studies on the role of genes involved in different functions [2], [1], [2].

The classical method of leaf cell counting requires the scanning electron microscope (SEM), but it is an expensive and time-consuming method and thus makes impractical the large-scale screening of epidermis [5].

Effective methods based on optical microscopy are also available [5,6]. Notwithstanding, such approaches require time-consuming operations such as soaking in ethanol in order to clear off chlorophyll by washing [6] and agarose-based epidermal imprinting or tape-based epidermis tearing depending on leaf type [5]. Moreover, the cell identification by means of image analysis is performed manually.

Here we describe a quick and simple method for counting leaf epidermal cells without leaf treatments and based on automatic cell marking. This method, previously used by authors of this paper for *Lotus japonicus* trifolia [2], was improved here by using an open-source software (ImageJ) and furtherly validated on *Oxalis pes-caprae* and *Viola odorata* leaves.

Instruments used by authors were Carl Zeiss Jena JENAPOL polarizing microscope and Nikon D300s SLR digital camera. The magnification of the lens was 20x.

## Declaration of Competing Interest

The authors declare that they have no known competing financial interests or personal relationships that could have appeared to influence the work reported in this paper.

## Appendix Supplementary materials

Image, application 1
