# Supplementary material for: Automatic cell identification and counting of leaf epidermis for plant phenotyping
Source: MethodsX. 2020 Mar 14;7:100860. doi: 10.1016/j.mex.2020.100860 (PMC7132086; doi:10.1016/j.mex.2020.100860)
Supplement: Supplementary file 1 [file mmc1.docx]

**Supplementary material**

The provided Supplementary figures (1-4) are micrographs that can be used to test the proposed method on three different plant species. The Supplementary figures 1 and 2 are two micrographs of the same species with different pixel size.


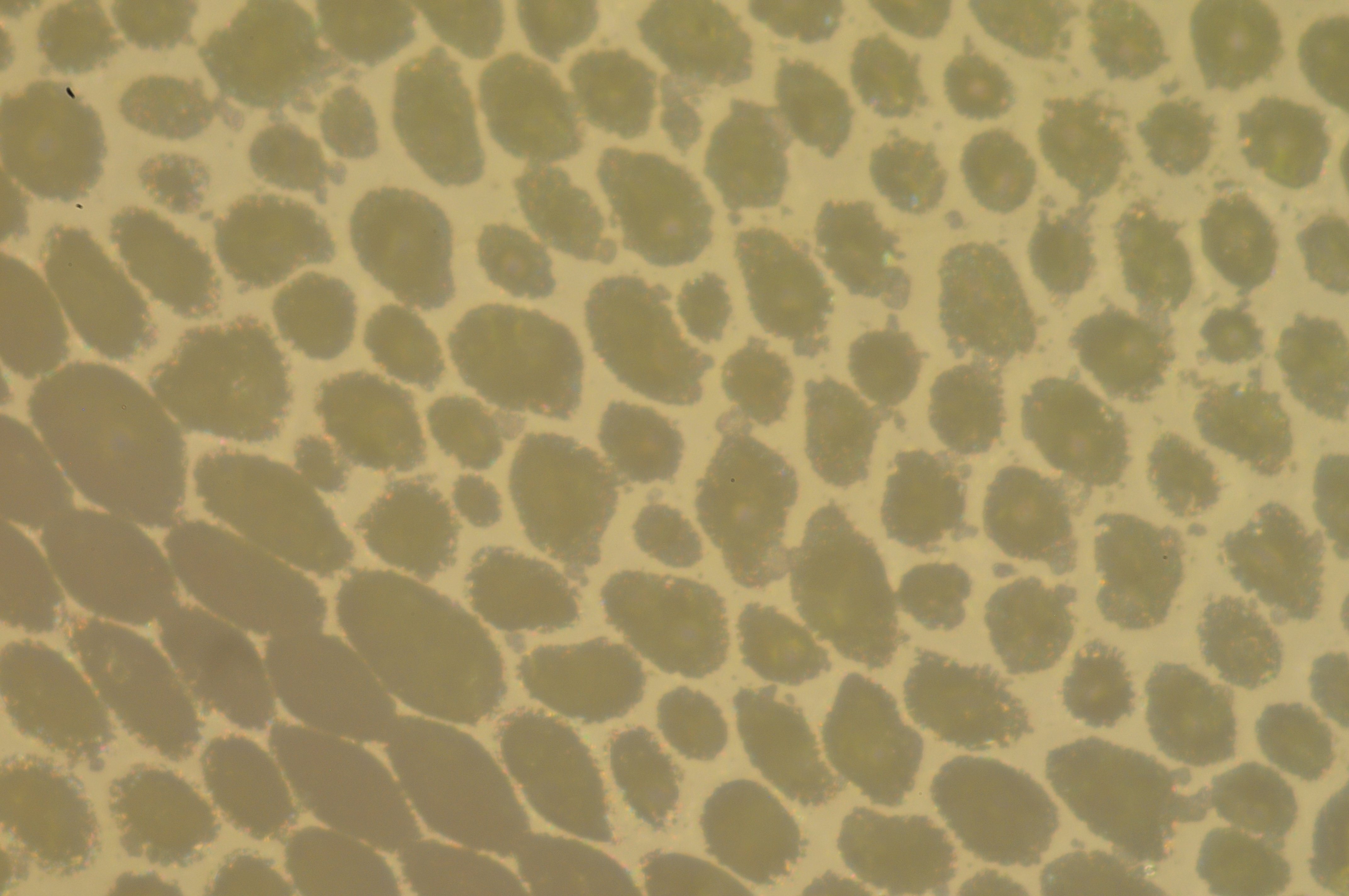


Supplementary figure 1. Example micrograph of *Lotus japonicus* leaf (pixel sixe 0.117 microns).


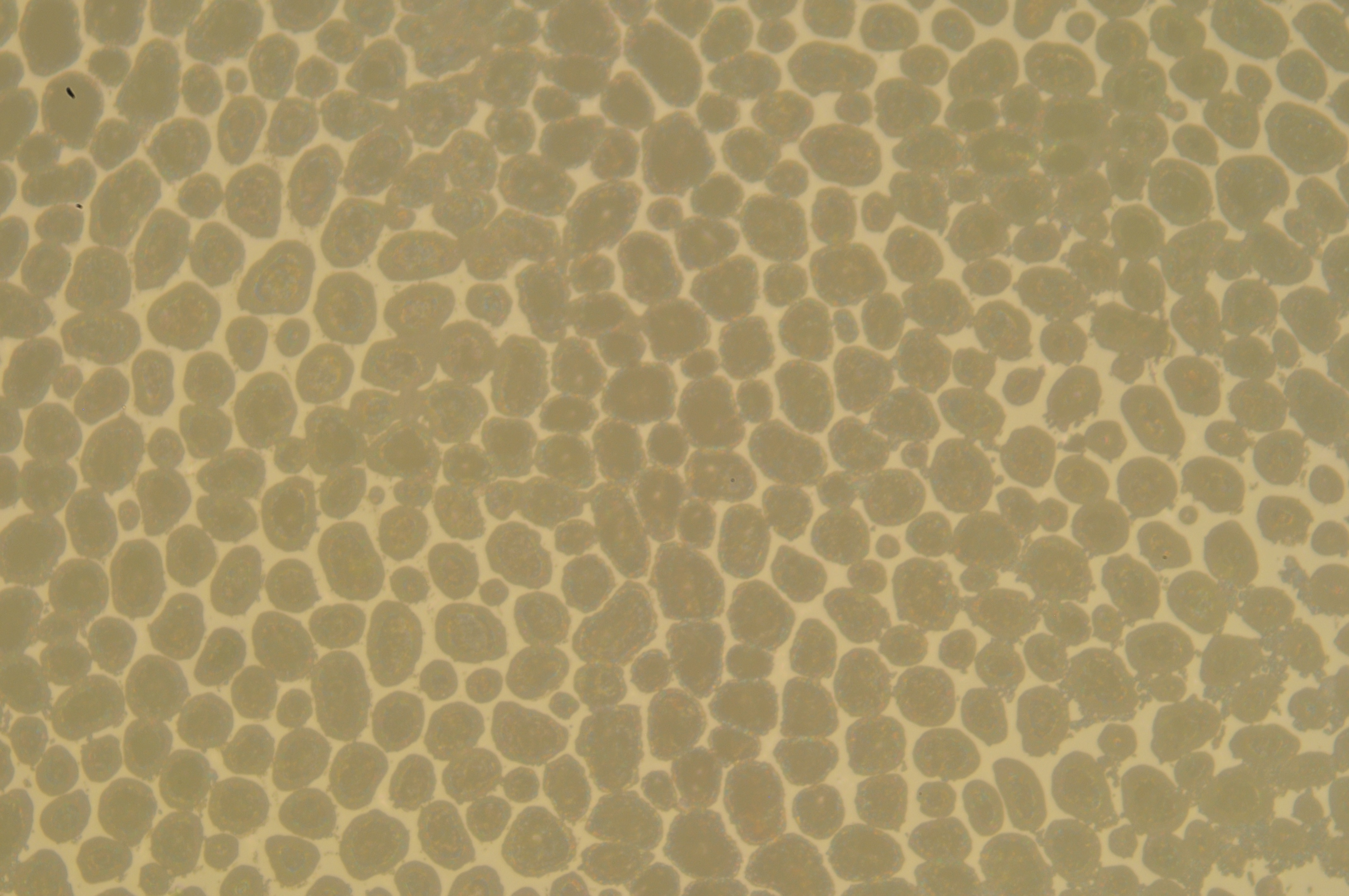


Supplementary figure 2. Example micrograph of *Lotus japonicus* leaf (pixel sixe 0.233 microns).


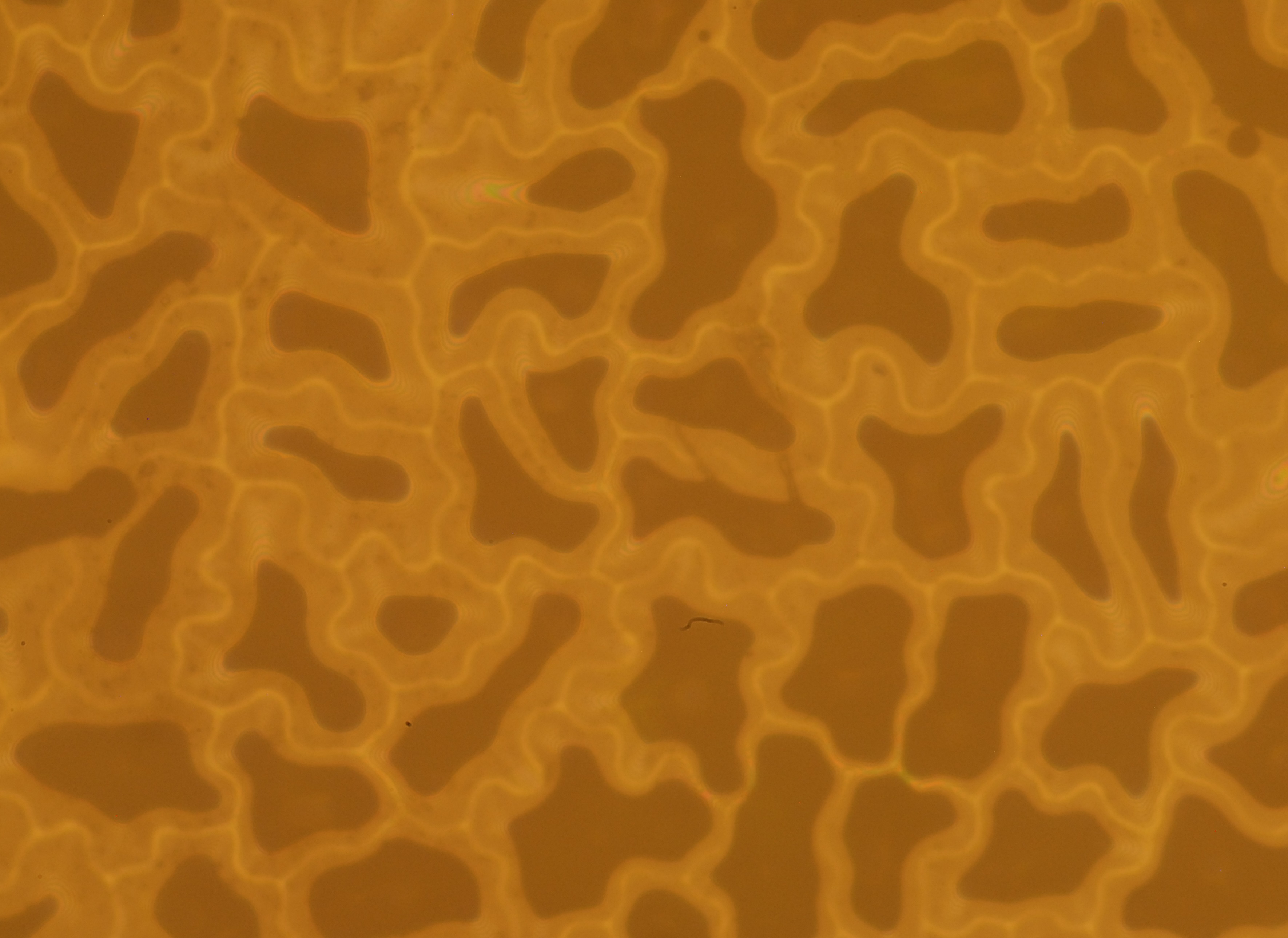


Supplementary figure 3. Example micrograph of *Oxalis pes-caprae* leaf (pixel sixe 0.130 microns).


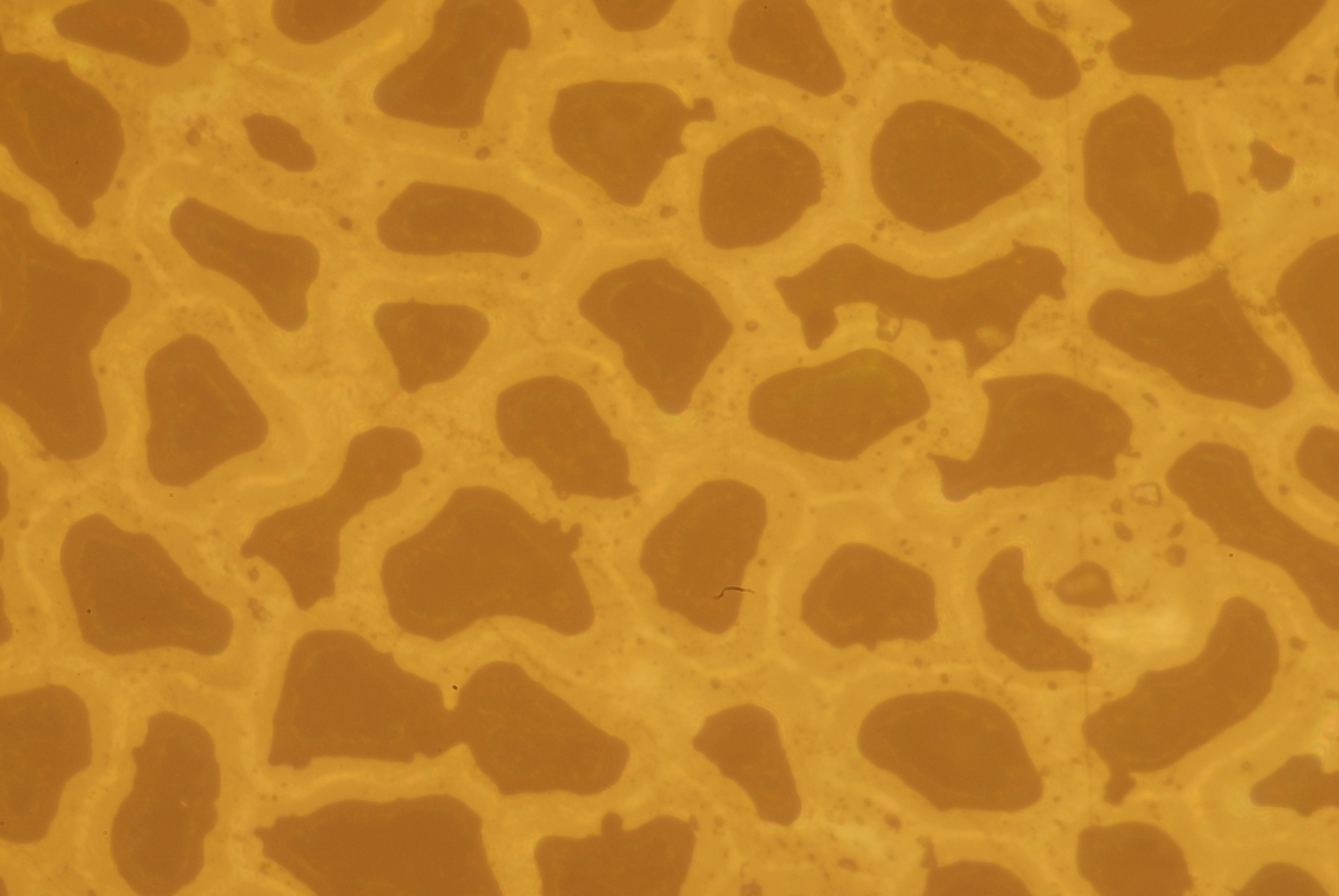


Supplementary figure 4. Example micrograph of *Viola odorata* leaf (pixel sixe 0.130 microns).


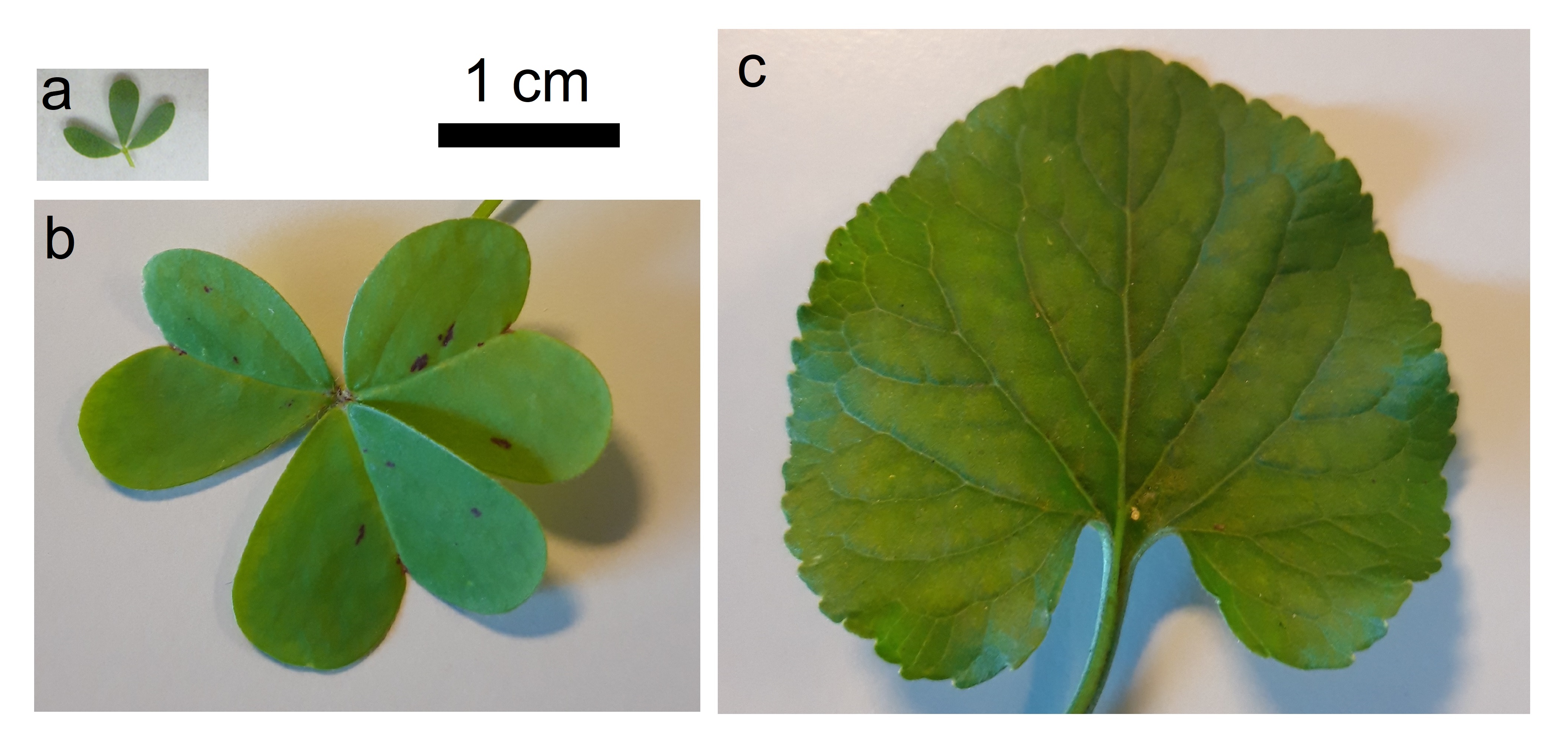


Supplementary figure 5. The leaves of the three different plant species (a) *Lotus japonicus,* (b) *Oxalis pes-caprae* and (c) *Viola odorata* used in the paper to test the method.
